# Genome-Assisted Gene-Flow Rescued Genetic Diversity Without Hindering Growth Performance in an Inbred Coho Salmon (*Oncorhynchus kisutch*) Population Selected for High Growth Phenotype

**DOI:** 10.1007/s10126-025-10416-1

**Published:** 2025-02-01

**Authors:** Junya Kobayashi, Ryo Honda, Sho Hosoya, Yuki Nochiri, Keisuke Matsuzaki, Koichi Sugimoto, Atsushi J. Nagano, Akira Kumagai, Kiyoshi Kikuchi, Tadahide Kurokawa

**Affiliations:** 1 Fisheries Laboratory, Graduate School of Agricultural and Life Sciences, University of Tokyo, 2971-4 Bentenjima, Maisaka, Hamamatsu, Shizuoka, 431-0214 Japan; 2 Miyagi Prefecture Fisheries Technology Institute, Freshwater Fisheries Experimental Station., Miyagi, Taiwa, 981-3625 Japan; 3 Faculty of Agriculture, Ryukoku University, Yokotani 1-5, Seta Ohe-Cho, Shiga , Otsu-Shi, 520-2194 Japan; 4 Institute for Advanced Biosciences, Keio University, 403-1 Nipponkoku, Daihouji, Tsuruoka, Yamagata, 997-0017 Japan; 5 Fisheries Resource Institute, Japan, Fisheries Research and Education Agency , Kushiro Field Station, 116 Katsurakoi, Kushiro, Hokkaido 085-0802 Japan

**Keywords:** Coho salmon, Genetic rescue, Genomic selection, Sustainable aquaculture

## Abstract

Selective breeding is a powerful tool for improving aquaculture production. A well-managed breeding program is essential, as populations can otherwise lose genetic diversity, leading to reduced selection response and inbreeding excesses. In such cases, genetic diversity in broodstock must be restored by introducing individuals from external populations. However, this can reduce the accumulated genetic gains from selective breeding. However, the selective introduction of individuals with superior phenotypes will allow the restoration of genetic diversity without sacrificing these gains. In this study, we demonstrated this possibility using a selectively bred (SB) and a randomly bred (RB) population of coho salmon (*Oncorhynchus kisutch*). Forty males with superior growth were selected from the RB population using genomic selection and crossed with 127 randomly collected females from the SB population, producing a newly bred (NB) population. Genetic diversity, assessed from population statistics such as effective number of alleles, allele richness, and observed heterozygosity of 11 microsatellite markers, was higher in NB than in SB and RB. Additionally, fork length and body weight were compared among the three populations after 12 months of growth post-fertilization in common tanks. The least-squares means of fork length and body weight were similar between NB (164.9 mm and 57.9 g) and SB (161.1 mm and 53.7 g), while both were significantly greater than RB (150.4 mm and 43.0 g). Our results highlight the effectiveness of genome-assisted gene flow in restoring the genetic diversity of a population without compromising accumulated genetic gain in growth.

**Supplementary Information:**

The online version contains supplementary material available at 10.1007/s10126-025-10416-1.

## Introduction

Aquaculture is an important industry that plays a critical role in securing food in today’s world, where the demand for food is increasing due to global population growth and changes in the global environment (FAO 2015. Meeting the sustainable development goals. Rome: FAO 2015). While there are various approaches to improving production efficiency, scientific selective breeding is one of the most powerful tools for increasing production (Gjedrem et al. 2012). For example, a great proportion of major aquaculture species such as farmed Atlantic salmon (*Salmo salar*), rainbow trout (*Oncorhynchus mykiss*), and Genetically Improved Farmed Tilapia (GIFT), are derived from selective breeding populations. To benefit from selective breeding a well-managed breeding program is required (Houston et al. 2022; Boudry et al. 2021). Otherwise, poor management, i.e., the use of a small number of broodstock and/or the use of close relatives only, will result in losses in genetic diversity leading to poor selection response, or inbreeding excess, necessitating termination or revisions of the program (Gjedrem and Baranski 2009; Gjedrem and Kolstad 2012; Huang and Liao 1990; Hulata et al. 1986; Kincaid 1983; Knibb et al. 2014; Telchert-Coddington and Smitterman 1988). This is especially true for breeding programs with small populations, and close attention to genetic diversity is required (Taberlet et al. 2007).

If a loss of genetic diversity exceeds the level required for sustainable management, the broodstock may require restoration of genetic variation. Restoration of genetic diversity can be partly achieved by increasing the number of broodstock. However, this approach cannot restore the alleles which have been lost due to genetic drift. On the other hand, the introduction of genetic material from an external population can increase the number of alleles, allele richness, and gene diversity (Gjøen and Bentsen 1997; Knibb et al. 2020). This is analogous to the genetic rescue or assisted gene flow applied in conservation programs (Aitken and Whitlock 2013; Kristensen et al. 2015; Ghildiyal et al. 2023; Pregler et al. 2023). Genetic rescue is the attempt to increase genetic diversity and mitigate the detrimental effects of inbreeding by introducing genetic resources from external populations. While the introduction of individuals from outside can increase genetic diversity, it can also reduce the fitness of the wild population (i.e., outbreeding depression) (Allendorf et al. 2001; Frankham et al. 2011) or the accumulated genetic merits of the selected population (Gjøen and Bentsen 1997). Recently, genetic rescue with the aid of genomics has been implemented (Allendorf et al. 2010; Carlson et al. 2014; Supple and Shapiro 2018; Theissinger et al. 2023; Schmidt et al. 2024). One such approach is the genomic prediction of adaptation and fitness-related phenotypes, or genomic selection (Meuwissen et al. 2001), that enables the selection of individuals better adapted to the introduced environments (Browne et al. 2019; Strandén et al. 2019). It is hypothesized that this approach is also effective in increasing the genetic diversity of small aquaculture populations without diminishing the genetic gain accumulated through selective breeding (Hosoya et al. 2018).

Coho salmon (*Oncorhynchus kisutch*) is one of the important aquaculture species ranked 4th in production in Japan (Statistical Survey on Marine Fishery Production, Ministry of Agriculture, Forestry and Fisheries, Japan, 2023: https://www.e-stat.go.jp/stat-search/files/data?sinfid=000040181768&ext=xls, EXCEL file in Japanese, accessed September 2024). This species is not native to Japan, and the domestic stock is maintained using a population imported from North America in the 1970s. Since then, broodstocks have been maintained at small scales. In 2011, the main production area of Japanese cultured coho salmon, i.e., Tohoku District including Miyagi Prefecture, was severely damaged by the tsunami disaster of the Great East Japan Earthquake (Sasaki et al. 2017). Immediately after the disaster, a national project was initiated to reconstruct the industry (“A scheme to revitalize agriculture and fisheries in disaster area through deploying highly advanced technology” funded by the Agriculture, Forestry, and Fisheries Research Council and Reconstruction Agency, Japan). This included the utilization of a coho salmon population with high growth phenotype produced by phenotypic selection, namely a selectively bred (SB) population, at the Inland Fisheries Experimental Station, Miyagi Prefecture Fisheries Technology Center (Miyagi, Japan). Meanwhile, the genetic health of the SB population has been assessed using genome-wide single nucleotide polymorphisms (SNPs) obtained by ddRAD-seq (Hosoya et al. 2018). The genetic analysis revealed reduced additive genetic variation and high genetic relatedness among individuals, suggesting the necessity of restoration of genetic diversity for the SB population.

In this study, we tested the possibility of a genome-assisted gene flow to restore the genetic diversity of the SB population without deteriorating its growth performance. Individuals with large breeding values for body size (fork length (FL) and body weight (BW)) were selected from the randomly bred (RB) population, the source population of the SB, using genomic prediction. These selected individuals were crossed with randomly collected SB individuals to create a new breed (NB). The genetic diversity and growth performance were compared among the three populations.

## Materials and Methods

### Ethical Disclosure

The phenotype recording and sample collection were carried out at the Freshwater Fisheries Experimental Station, Miyagi Prefecture Fisheries Technology Institute (Miyagi, Japan). The experiment was approved by the Miyagi Prefectural Evaluation Committee for Research and Development Institutes.

### Selection of Broodstock from the Randomly Breeding (RB) Population

The origin of the coho salmon populations (SB and RB) was described by Hosoya et al. (2018). The original population was introduced from the Lower Kalama hatchery (WA, USA) to Japan in 1978 and a subpopulation was transferred to the station in 1980. The population was subsequently maintained without individual or family identification until 2000. The selection program started with a very small number of broodstock: 16 females that constituted the top 10% for BW at maturation were crossed with 13 randomly chosen males in 2000. The descendants were subjected to the second phenotypic selection in 2003 where 40 females and 10 males consisting with the greatest BW were crossed. Subsequently, random crosses were done in 2006 using 165 females and 33 males, in 2009 using 71 females and 23 males, and in 2013 using 104 females and 91 males (hereafter referred to as SB-YC13). The RB population used in this study was maintained separately from the SB population. The number of broodstock of the RB population varied per generation according to the financial condition for the year: 142 females and 13 males in 2000, 52 females and 18 males in 2003, 136 females and 43 males in 2006, 40 females and 16 males in 2009, 64 females and 51 males in 2013 (RB-YC13). Before 2013, the crossing pattern was 10 to 15 females were crossed with 3 to 5 males, and this set was repeatedly created. There were no individuals reused across sets. At the cross in 2013, 5 to 15 females were crossed with a similar number of males, and this set was repeatedly created without being reused across sets.

Judging from morphological observation at sampling (i.e., 33 months old), we anticipated that few RB females would reach maturity during the year. Thus, we decided to cross RB males and SB females to produce NB. At 33 months old (September 2016), we selected RB-YC13 individuals (*n* = 768) with male-like morphology and measured the FL and BW of each fish. The average (± standard deviation) FL and BW were 26.4 (± 3.0) cm and 222.5 (± 76.5) g, respectively (Supplementary Table S1). A fin clip was collected from the caudal fin for each specimen, and genomic DNA was extracted from the clip using a Gentra Puregene Tissue Kit (QIAGEN, Venlo, Netherland) following the manufacturer’s instructions. The DNA concentration was measured using a QuantiFluor dsDNA kit (Promega) and adjusted for 70 ng/uL using milli-Q water. The genetic sex was examined for each individual using a male-specific primer (OTY2-WSU) following Brunelli and Thorgaard (2004). The PCR amplification was done using a T100 thermal cycler (Bio-Rad). Fragment analysis was done using an Applied Biosystems 3130 Genetic Analyzer (Thermo Fisher). Male-specific amplicon (435 bp) was detected from 515 out of 768 individuals. The biased sex ratio was observed because we collected individuals who showed male-like morphology at sampling. Among the 515 genetic males, 300 fish were randomly chosen for subsequent ddRAD-seq. We set this sample number based on the financial limitations and the throughput of a single lane of TruSeq v3 chemistry (PE100) on an Illumina HiSeq 2000 platform used for the subsequent sequencing. The library construction was done following Sakaguchi et al. (2015) where BglII and EcoRI (TOYOBO, Osaka, Japan) were used for DNA digestion.

An average of 829,797 paired reads were obtained with the range of 40,049 to 2,992,012 (Supplementary Table S2). All sequence data is registered with the DDBJ (BioSample Submission ID: SSUB031092, available after publication). Sequence data are available at DDBJ DRA (accession ID: DRA019425). Adapter sequences were trimmed using Trimmomatic-0.39 (Bolger et al. 2014) with the following threshold: allowing 2 mismatches, palindrome clip threshold of 30, and simple clip threshold of 10 (i.e., ILLUMINACLIP:TruSeq3-PE-2.fa:2:30:10); remove reads with leading quality less than 20 (LEADING:20); remove reads with trailing quality less than 20 (TRAILING:20), minimum length of 50 bp (MINLEN:50). The read pairs remaining at both sides were mapped onto the Coho salmon reference genome (Okis_V1; GenBank assembly accession: GCF_002021735.1) using BWA-mem (BWA v0.7.15) (Li 2013). Subsequently, reads with the flag of 4 (unmapped), 256 (not primary alignment), or 2048 (supplementary alignment) and those with mapping quality less than 10 were excluded using Samtools v 1.3.1 (Li et al. 2009) (i.e., *-F 2308 -q 10*).

Genotype calling was performed using FreeBayes (v1.0.2) (Garrison and Marth 2012). Subsequently, only biallelic SNPs with minor allele frequency larger than 0.02 were extracted and SNPs with high missing rate (> 40%) were excluded using VCFtools (v0.1.14) (Danecek et al. 2011). These per-chromosome genotype files were concatenated using BCFtools concat (v1.3.1) (Li 2009; Danecek et al. 2021). Additionally, genotypes with low (< 8) and high (> 300) depth of coverage were masked and the missing rate filter was applied again (i.e., retained SNPs with the maximum missing rate of 40%). Finally, individuals with a genotype call rate of less than 85% were excluded. The final number of individuals was 198 and the number of SNPs was 5,929. While the SNP size is enough for breeding value prediction for aquaculture species (Kriaridou et al. 2020; Hosoya et al. 2021), the small sample size will reduce prediction accuracy. However, relatively small numbers of samples are required for aquaculture breeding (Zenger et al. 2019), and precise prediction was not pursued in this study as we did not aim for continuous selective breeding. In addition, we selected broodstock candidates based on both breeding value and maturity status. Therefore, a limited sample size does not pose a problem in this study.

Genomic estimated breeding value (GEBV) was estimated for FL and BW at 33 months of age using the genomic best linear unbiased prediction (GBLUP) model per phenotype:

$${\varvec{y}}={\varvec{\mu}}+{\varvec{Z}}{\varvec{a}}+{\varvec{e}}$$

Where ***y***, ***μ***, and ***e*** are the vector of observed phenotype, the phenotypic means, and the residuals, respectively; ***Z*** is the corresponding incidence matrices for the additive effects ***a***, which follow a normal distribution ~ ***N***(0, ***Gσ***_***a***_^***2***^), where ***G*** is the genomic relationship matrix and ***σ***_***a***_^***2***^ is the genetic variance. We assume the residuals follow a normal distribution ~ ***N***(0, ***Iσ***_***e***_^***2***^), where ***I*** is an identity matrix and ***σ***_***e***_^***2***^ is the error variance. The ***G*** matrix was obtained with the *A.mat* function and the GBLUP model was solved using the *kin.blup* of the R package rrBLUP (4.4) (Endelman 2011). Narrow sense heritability was determined as ***h***^*2*^ = ***σ***_***a***_^***2***^ /(***σ***_***a***_^***2***^ + ***σ***_***e***_^***2***^). We selected 40 individuals based on the rank of GEBVs, i.e., the sum of the GEBVs divided by the average for each phenotype, and crossed with 127 females randomly sampled from SB (SB-Y13) to create a new breed (NB-Y16) in December 2016. Each male was mated with three or four females. There was no overlap of mates between males. Note that these 40 sires are not necessarily the top 40 individuals, as some of the top individuals did not reach maturity at mating and some others died before the breeding season (Supplementary Table S3). A new generation of SB (223 dam × 110 sire) and RB (37 dam × 40 sire) was created at the same time (referred to as SB-Y16 and RB-Y16, respectively). The number of broodstock for RB-Y16 was smaller than for SB-Y16 because the number of matured individuals was smaller in RB-Y13 than in SB-Y13. At production, 5 to 15 females were crossed with similar numbers of males, and this set was repeatedly created without overlapping. At the swim-up stage (February 2017; 3 months post-fertilization), approximately 100 individuals from each of the three populations were sampled to examine the genetic diversity, and the rest were mixed in a communal holding tank for growth comparison.

### Assessments of Genetic Diversity and Differences Among Three Populations

Sampling for genetic diversity was done using 11 microsatellite markers designed in previous studies (Beacham et al. 2011; Khoo et al. 2000; Nelson et al. 1998; Palti et al. 2002; Rexroad et al. 2002; Rodriguez et al. 2003; Sakamoto et al. 1996; Scribner et al. 1996; Smith et al. 1998; Williamson et al. 2002) (Supplementary Table S4). The sample numbers were; SB-Y16 = 95, RB-Y16 = 96, and NB-Y16 = 100. Genomic DNA was collected from an adipose fin clip using a Gentra Puregene Tissue Kit (QIAGEN). The 11 loci were amplified using the tailed primer method (Schuelke 2000; Sekino et al. 2006) where 19-bp of the M13 sequence (CACGACGTTGTAAAACGAC) was added to the 5' end of the forward primer to label PCR amplicon with a fluorescent dye (i.e., FAM, VIC, NED, PED). Polymorphisms were analyzed on an ABI3130 DNA Analyzer (Applied Biosystems, MA, USA). The PCR conditions for each marker are described in Supplementary Table S5.

The allele richness (*A*_R_) was obtained using the *allel.rich* function of R package PopGenReport ver 3.0.0 (Adamack and Gruber 2014), and the number of effective alleles (*A*_E_) was calculated as 1/$\sum_{i=1}^{n}{p}_{i}$ were *p*_*i*_ is the frequency of each allele and *n* is the number of alleles at the locus. Observed heterozygosity (*H*_O_), mean gene diversities within population (*H*_S_), and fixation index (*F*_IS_) were obtained using the *basic.stats* function of R package hierfstat (ver0.5–11) (Goudet 2005). In addition, pair-wise *F*_*ST*_ (Weir and Cockerham 1984) and its 95% confidence interval were calculated using the *genet.dist* and the *boot.ppfst* (the number of bootstraps = 100) functions of hierfstat, respectively.

Discriminant analysis of principal components (DAPC) was conducted to assess the population structure using the *dapc* function of the R package adegenet (ver2.1.10) (Jombart 2008); the number of axes retained in the Principal Component Analysis (PCA) step was set at 60 (*n.pca* = 60) and the number of axes retained in the Discriminant Analysis step was 2 (*n.da* = 2). The scatter plot was drawn using *scatter* function of adegenet. We also conducted STRUCTURE analysis (Hubisz et al. 2009) assuming the number of max populations as 2 (*MAXPOPS 2*) and no admixture.

### Growth Comparison Test

FL and BW of individuals reared in the communal tanks were compared among populations at 12 months post-fertilization (November 2017). We note that body size at this age (12 months) may not be the same trait as that at 33 months old when the phenotypes for GEBV prediction were collected. Thus, the body size comparison should also have been made on 33-month-old fish. However, we could not continue this experiment until this age due to the capacity of the facility and the duration of the research grant (ended March 2018).

After 9 months post-fertilization (August 2017), a total of 737 individuals were transferred into five 1-kL circular tanks (148, 149, 143, 148, 149 fish per tank). Each individual was tagged with a passive integrated transponder (PIT) tag, and the adipose fin-clipped for genomic DNA extraction. FL and BW were recorded. These fish were then returned back into their original tank. They were reared for another 3 months (until November 2017) and FL and BW were again recorded. At this sampling point, 15 fish were lost due to mortality or tag drop-off, resulting in 722 specimens used for the assignment test.

### Population Assignments

As the genetic origin of the 722 individuals used in the growth comparison test was unknown, their population ID was inferred from the 11 microsatellite markers. Genomic DNA extraction and genotyping of the 11 loci were done as described above. We used NewHybrid software (Anderson and Thompson 2002) with an initial 20,000 burn-in and subsequent 50,000 post burn-in sweeps. The genotype data used for the genetic diversity analysis (i.e., the 291 individuals with known population origin) was included as the training set. The accuracy of population assignments was evaluated from the following three aspects. First, the proportion of the individuals accurately assigned to the original population. Second, correlations in allele frequencies between the inferred population (infRB: inferred as RB-Y16, infSB: inferred SB-Y16, and infNB: inferred NB-Y16) and the training sets (RB-Y16, SB-Y16, and NB-Y16, respectively) were assessed per locus using Spearman’s correlation coefficient (ρ). Finally, pairwise Weir and Cockerham *F*_ST_ were calculated among the six groups.

### Statistical Analysis for Growth Comparison

Population difference in body size at 12 months post-fertilization was examined using a general linear mixed model (LMM). The model was:

$$y = Xb + Za + e$$

Where ***y***, ***b***, and ***a*** are vectors of phenotype, fixed population effects, and random tank effects, respectively; ***X*** and ***Z*** are the design matrix for ***b*** and ***a***; ***e*** is a vector of residuals. The equation was solved using the *glm* function of R/stats. Model comparison was done among models (i.e., with and without the population effects) based on Akaike’s information criterion (AIC) (Akaike 1973). The least squares mean for each population was compared using the *emmeans* function of the R package emmeans (ver. 1.7.2) (Lenth 2022). The significant threshold was set as *p* < 0.05 and *p*-value were adjusted using the Bonferroni method.

## Results

### Genomic Selection from the RB-Y13

**Fig. 1:**
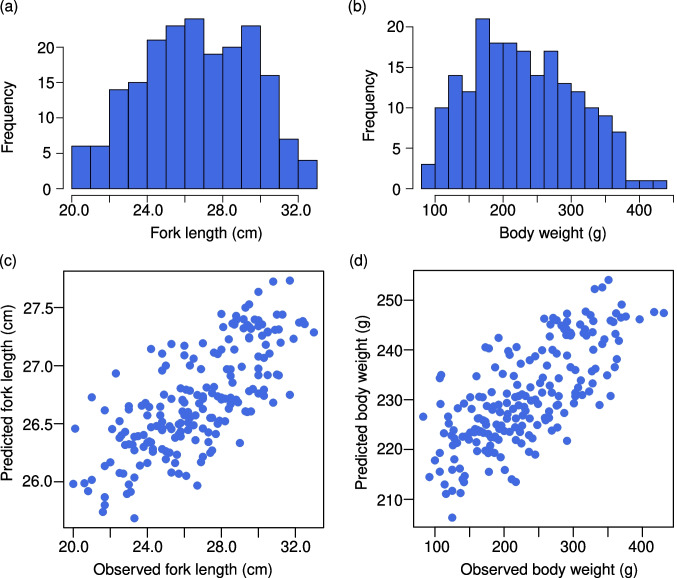
Histogram of fork length (**a**) and body weight (**b**), and correlation between observed and predicted values for fork length (**c**) and body weight (**d**) for RB-Y13

Individual selection from the RB-Y13 was done using the GBLUP method (198 males and 5,929 SNPs). The average FL and BW (± standard deviation) of these individuals were 26.7 (± 2.9) cm and 230.5 (± 76.4) g, respectively. The distribution of these phenotypes was not normal (Shapiro–Wilk test: *p* = 0.034 and 0.005, respectively) (Fig. 1a, b) partly because the initial sampling of the 768 individuals was not random but chose individuals with male-like morphology. Thus, smaller individuals were not involved. Reflecting this, at least partially, the estimated heritability was low for both FL and BW (*h*^*2*^ = 0.119 and 0.103, respectively) and the correlation coefficients between observed phenotype and GEBV were moderate (*r* = 0.731 and 0.727, respectively) (Fig. 1c, d). We ranked the 198 males based on the sum of GEBVs divided by the average for FL and BW and selected 40 matured individuals from the top rank (Supplementary Table S1).

### Restoration of Genetic Diversity in NB-Y16

To assess the restoration of genetic diversity in NB-Y16, we genotyped 11 microsatellite markers and compared genetic parameters among RB-Y16, SB-Y16, and NB-Y16. Genotype information of each individual is available in Supplementary Table S6. The mean values of the number of alleles (*A*_N_), allele richness (*A*_R_), the number of effective alleles (*A*_E_), observed heterozygosity (*H*_O_), mean gene diversities within population (*H*_S_), and fixation index (*F*_IS_) are listed in Table 1 (detailed information is available in Supplementary Table S2). As expected, SB-Y16 had smaller values for these statistics than RB-Y16, while these values were highest in NB-Y16, the admixed population between SB and RB. This result clearly shows a restoration of genetic variation by introducing individuals from external populations. The only exception was *F*_IS_, where the value was smaller in SB than in RB. It is expected that the higher the *F*_IS_, the higher the genetic relatedness of the parental individuals. However, *F*_IS_ does not necessarily represent the genetic diversity at the population level. Indeed, the value of RB-Y16 is close to zero while *H*o was the smallest in SB-Y16. The negative *F*_IS_ of NB-Y16 indicates heterozygote excess. This was in line with the expectation as NB-Y16 was produced between the two genetically isolated populations.

**Table 1:** Mean values of genetic statistics averaged over 11 microsatellite markers

	*AN*	*AR*	*AE*	*HO*	*HS*	*FIS*
RB-Y16 (*n* = 95)	6.91	6.74	3.98	0.715	0.72	0.008
SB-Y16 (*n* = 96)	6.18	6.13	3.41	0.67	0.648	− 0.033
NB-Y16 (*n* = 100)	7.73	7.53	4.25	0.778	0.723	− 0.076

*AN* number of alleles, *AR* allele richness, *AE* number of effective alleles, *HO* observed heterozygosity, *HS* mean gene diversities within population, *FIS* fixation index

### Extent of Genetic Differentiation Among the Three Populations

We evaluated the extent of genetic differentiation among populations using pair-wise Weir Cockerham *F*_ST_ (Table 2). Significant genetic differentiation was observed in each pair, but the extent of differentiation was smaller between NB-Y16 and RB-Y16 (0.046) or SB-Y16 (0.041), compared to the RB-Y16 and SB-Y16 pair (0.135).

**Table 2:** Pair-wise Wier Cockerham FST with lower and upper boundary of 95% confidence interval in parentheses

	Pair-wise FST
RB-Y16–SB-Y16	0.135 (0.103–0.180)
RB-Y16–NB-Y16	0.046 (0.030–0.061)
SB-Y16–NB-Y16	0.041 (0.031–0.055)

**Fig. 2:**
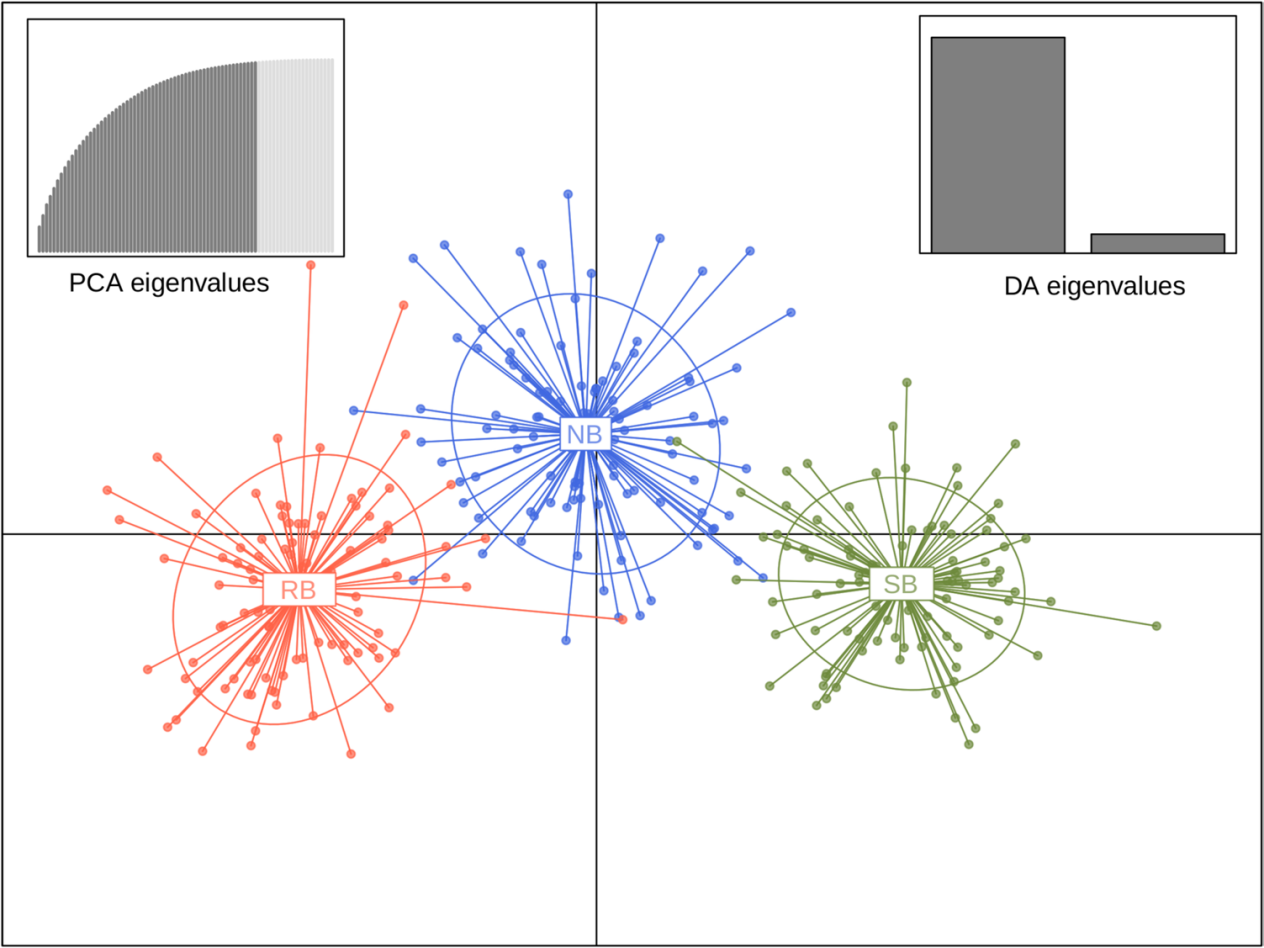
DAPC scatter plot. The *X*- and *Y*-axes are the 1st and 2nd principal components of DAPC, respectively. Each point represents individuals of RB (red), SB (green), and NB (blue) populations. The insets on the top-right and top-left show scree plots of PCA and DA eigenvalues, respectively

**Fig. 3:**
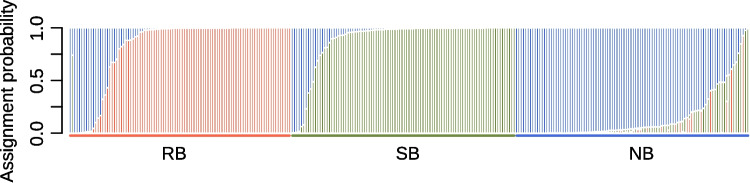
Structure assignment plots. Each vertical bar indicates cumulative assignment probability for RB (red), SB (green), and NB (blue) populations for each individual (RB: *n* = 95, SB: *n* = 96, NB: *n* = 100)

Genetic differentiation was also confirmed from DAPC analysis (Fig. 2). While RB-Y16 and SB-Y16 populations were clearly separated, the NB-Y16 population was plotted between the two populations with some extent of overlap at the individual level. The STRUCTURE analysis also revealed genetic differentiation at the population level (Fig. 3). In line with results from DAPC analysis, some individuals exhibited a greater degree of shared genetic ancestry with those from the different populations than with the original population.

### Population Assignments

The genetic origin of the 722 individuals used for the growth comparison test was inferred from the 11 microsatellite markers with the training set (a total of 291 individuals) that consisted of individuals with genetic origin used in the genetic differentiation analysis described above. These individuals were evenly assigned to the three populations (infRB = 243, infSB = 248, infNB = 231) (Supplementary Table S7). The accuracy of the assignment was assessed using the training set. Within the training set, 1 and 3 individuals from RB were misassigned to SB and NB, respectively, while 3 individuals of SB were misassigned to NB (Table 3). Meanwhile, 6 and 4 individuals of NB were misassigned in RB and SB, respectively. The overall accuracy of population assignments for the training set was 94.2% (274 out of 291): RB = 95.8%, SB = 96.8%, NB = 90.0%. These misassignments reflected the genetic variation within each population, as observed in the genetic differentiation analysis.

**Table 3:** Summary of the results from population assignments

	Sample number	Assigned population			Accuracy
		RB-Y16	SB-Y16	NB-Y16	
RB-Y16	95	91	1	3	0.958
SB-Y16	96	0	93	3	0.969
NB-Y16	100	6	4	90	0.900
Unknown	722	243	248	231	–

The accuracy of population assignments was also assessed using allele frequency. The Spearman’s correlation coefficient (*ρ*), calculated for allele frequencies between each population pair (i.e., infRB–RB-Y16, infSB–SB-Y16, and infNB–NB-Y16), ranged from 0.762 to 1.000, and mean values were 0.938, 0.950, and 0.936, respectively (Table 4). There was a locus (Oneu13) at which the correlation coefficient in infRB and RB-Y16 was exceptionally small, but the reason for this is not clear. We also examined the degree of population differentiation among the six groups (RB-Y16, SB-Y16, NB-Y16, infRB, infSB, and infNB). Between the two sibling pairs, *F*_ST_ values were nearly zero, although the 95% CI was larger than zero between the infRB and RB-Y16 pair (0.001–0.009) (Table 5). When *F*_ST_ values between non-sibling pairs were compared, the inferred population and their sibling group showed similar values (e.g., RB-Y16 and SB-Y16 = 0.135, RB-Y16 and infSB = 0.130). These results indicate that inferred populations and their sibling groups had highly similar allele frequencies. Altogether, it can be said the accuracy of the population assignments was high.

**Table 4:** Correlation coefficient (Spearman’s ρ) between allele frequencies in the inferred population and it’s sibling group

Locus name	infRB–RB-Y16	infSB–SB-Y16	infNB–NB-Y16
OtsG249	0.937	0.972	0.918
Ots103	0.969	0.940	0.936
OtsG68	0.957	0.949	0.933
Oki1	1.000	0.829	0.943
Oneu13	0.762	0.948	0.948
Omm1058	0.938	0.938	0.980
Oki101	0.943	0.943	0.943
OmyRGT10TUF	0.893	1.000	0.857
OmyRGT39TUF	1.000	1.000	1.000
Omm1333	0.979	0.966	0.933
Omm1384	0.938	0.970	0.905
Mean	0.938	0.950	0.936

**Table 5:** Pair-wise FST (lower triangle) with 95% confidence interval (upper triangle)

	RB-Y16	SB-Y16	NB-Y16	infRB	infSB	infNB
RB-Y16	–	0.096–0.186	0.067–0.029	**0.001–0.009**	0.092–0.181	0.066–0.030
SB-Y16	0.135	–	0.027–0.055	0.089–0.157	** − 0.001–0.002**	0.027–0.049
NB-Y16	0.046	0.041	–	0.022–0.045	0.023–.054	** − 0.002–0.000**
infRB	**0.005**	0.118	0.03	–	0.000–0.152	0.022–0.046
infSB	0.13	**0.000**	0.037	0.113	–	0.022–0.046
infNB	0.045	0.038	** − 0.001**	0.032	0.033	–

Bold font indicates the results obtained between the inferred population and its sib-groups

### Growth Comparison Test

**Fig. 4:**
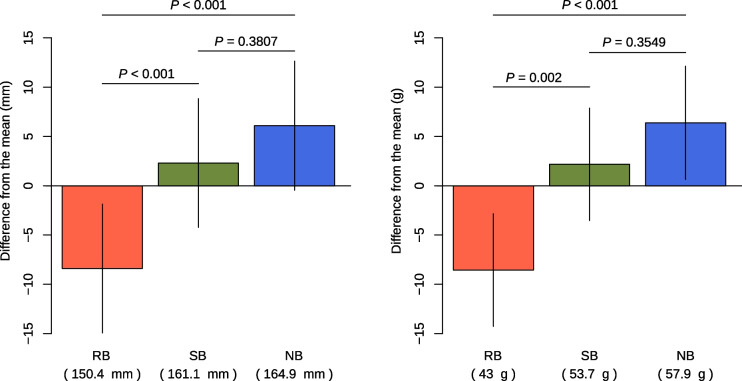
Least square mean of fork length (left) and body weight (right) of each inferred population. Vertical segments represent a 95% confidential interval. The values on the horizontal lines show adjusted *p* values for each comparison

The effect of selective introgression from RB into SB was evaluated by comparing body size at 12 months post-fertilization among the three inferred populations. At first, the significance of the population effect was tested using AIC. The AIC value of the full model was smaller than that of the model without population effect for both FL (AIC with and without population effect were 6817.529 and 6932.089, respectively) and for BW (with: 6856.331, without: 6967.426, respectively), indicating the significance of population effect. The least-square mean for FL and BW of infSB (FL = 161.1 mm, BW = 53.7 g) was significantly larger than those of infRB (150.4 mm, 43.0 g) (*p*-value < 0.0001 for FL and 0.0002 for BW, respectively) (Fig. 4). The mean values for these phenotypes of infNB (FL = 164.9 mm, BW = 57.9 g) was significantly larger than that of infRB (*p*-value < 0.0001 for both traits) while slightly, but not significantly, larger than that of infSB (*p*-value = 0.3807 and 0.3549, respectively), suggesting that the selective introgression did not deteriorate (or improve) the growth of the target population (i.e., SB) at 1 year post-fertilization.

## Discussion

The goal of this work was to increase the genetic diversity of the SB population, selected for body size at maturation by means of phenotypic selection, without losing its advanced growth phenotype. In this light, we have conducted selective introgression from an external RB population into the SB by creating the NB population. Increased genetic diversity was observed in the NB population while its growth performance, evaluated at 12 months post-fertilization, was similar to that observed in the SB population. These results indicated the efficiency of the selective introgression, although body size should have been compared at 33 months post-fertilization to confirm the effect of selection. The size differences measured at different ages could be manifesting different traits.

The validity of genetic rescue is now widely recognized (reviewed in Bell et al. 2019; Clarke et al. 2024; Fitzpatrick et al. 2023; Ralls et al. 2018). A potential risk of genetic rescue is outbreeding depression, where the overall fitness of the hybrids decreases due to reduced adaptability to the local environment. Additionally, co-adaptive gene complexes could be disrupted, such as gene–gene epistatic interaction, and/or gene–gene incompatibility (e.g., mito-nuclear incompatibility) (Edmands 2007; Hirase et al. 2020; Loope et al. 2024; Oakley et al. 2015). However, outbreeding depression is predictable and may not be as detrimental as previously thought (Frankham 2015; Pérez-Pereira et al. 2022; Clarke et al. 2024). Advanced genomic resources are now more accessible and will facilitate efficient genetic rescue and assisted gene flow as these tools enable pre-selection of source populations or individuals for introduction and allow monitoring/predicting the genomic consequences of hybridization (Aitken and Whitlock 2013; Browne et al. 2019; Fitzpatrick and Funk 2019; Capblancq et al. 2020; Hohenlohe et al. 2020; Chen et al. 2021; Guhlin et al. 2023). In livestock and poultry breeding, crossbreeding between large mainstream breeds and small local breeds is widely practiced to combine the economic advantages of mainstream breeds with the locally adaptive traits of small local breeds (landraces) (Biscarini et al. 2015; FAO 2015). Gene flow from mainstream breeds is often employed to improve economic traits and to enhance the genetic diversity of small local breeds, which frequently face challenges such as inbreeding and extinction. However, this can lead to genetic dilution and loss of genetic originality (Berthouly-Salazar et al. 2012; Giovannini et al. 2024; Kohl et al. 2019; Senczuk et al. 2020; Slagboom et al. 2022). Therefore, careful management of genetic diversity both within and between breeds is essential, and advanced genomic tools are valuable for achieving this balance (Phocas et al. 2015). In aquaculture breeding, while the application of genomic information to the genetic rescue of inbred populations and assisted gene flow remains limited if practiced at all, genomic information has been utilized to monitor the genetic diversity of cultured populations (Hosoya et al. 2018; Kajungiro et al. 2019; Longo et al. 2024; Palaiokostas et al. 2022; Sawayama et al. 2024). As we have demonstrated in this study, such information will be useful for the pre-selection of broodstock and planning breeding strategies for the conservation of populations at risk.

The effect of introgression on the restoration of genetic diversity of the recipient population can be increased when individuals are introduced from a genetically diverse population (Frankham 2015). As we selected individuals from the source RB population (RB-Y13) based on breeding values of body size trait and maturation, these individuals could have similar genotypes with the recipient SB population. In addition, the target trait for selection was the same as that used for the recipient SB population, and the source and the recipient populations were isolated only four generations ago. Therefore, the selected individuals might not have been genetically distant from the recipient population which can hinder the efficiency of introgression. Judging from the recovery in genetic statistics in the NB population, however, the selected individuals were genetically different from each other and from the SB individuals. Genetic relationships among selected individuals were traditionally estimated from pedigree information. Currently, molecular markers facilitate this estimation even for populations where pedigree information is unknown, such as the case of our populations (Hosoya et al. 2018). This information can assist in selecting individuals with wider genetic variation and optimize the balance between genetic improvements and the genetic diversity of selected individuals (Sonesson et al. 2012). In this study, the genome-wide SNP information used for genomic prediction could be used for the genetic relationship estimation. However, we did not consider genetic relationships for selection mainly because the number of candidate fish (*n* = 198) was small and there was little space to consider them for selection.

In this study, we could use a very small number of individuals to train the prediction model or the size of the training set, due to financial constraints. This raises a concern about the breeding value prediction, as accurate prediction requires large numbers of individuals (Goddard 2009). A relatively small training set may be sufficient for aquaculture species in practice due to the small effective population size (Zenger et al. 2019). Although better prediction can be achieved with the training set with a larger training set, training sets with a few hundred individuals are still available for genomic selection (Palaiokostas et al. 2016; Hosoya et al. 2018, 2021; Lin et al. 2020; Griot et al. 2021). In this study, we did not aim for precise breeding value predictions. Instead, we were more interested in selecting individuals with higher breeding value rankings from the training set. In addition, we also considered maturity for selection. Thus, we deemed it sufficient to observe a moderate correlation between predicted breeding values and phenotypic values (*r* = 0.731 for FL and 0.727 for BW, respectively), although a larger sample size could have yielded better rankings. Judging from the result that the mean body size of the NB individuals was comparable with that of the SB individuals, it can be said that the prediction and selection were effective, at least partially. If we used more individuals for selection, we could have accurately selected individuals with high breeding value and taken into account the genetic relationship between them, resulting in greater genetic diversity of the NB population.

Although the age at which the selection was made (33 months post-fertilization) and the age at which the growth comparisons were made (12 months post-fertilization) were different, our results suggest the potential of selective gene flow to restore genetic diversity of the introduced population without compromising the growth performance in the recipient population. One possible factor that allows NB to retain the same growth potential as SB is outcross enhancement, or hybrid vigor, also called heterosis. Here, offspring exhibit phenotypes superior to those of the parental populations. Hybrid vigor has been observed after the introduction of an external population for the purpose of genetic rescue by relieving inbreeding depression (Pickup et al. 2013; Robinson et al. 2017; Clarke et al. 2024). In aquaculture systems, hybrid vigor within species will be realized as non-additive genetic effects, explained by additive-additive epistasis (Joshi et al. 2020). In this study, the body size of the relatively inbred SB population was significantly larger than that of the RB population, and the NB population’s body size was comparable to that of the SB population. Therefore, it is considered that additive-additive epistasis (or hybrid vigor) is less likely to be the major factor in the NB population's body size being larger than the average of the two populations; however, it may have had a partial influence. This possibility will be tested by comparing the growth performances of the NB and a hybrid population produced between randomly chosen SB and RB individuals.

A limitation of this study is that growth performance was only assessed in the first generation, and there is no guarantee that this performance will be maintained at the same level in future generations. This is especially true if epistasis had a positive effect on the growth of NB. To sustain growth performance for generations, truncation selection may be needed to eliminate smaller individuals from broodstock. In this case, care must be taken to prevent loss of genetic variation. Another limitation is the uncertainty surrounding whether similar results would be achieved if the growth performance disparity between the selected and source populations was much greater than what we observed in this study. The success of genome-assisted gene flow depends on many factors and may not always restore genetic diversity without sacrificing accumulated genetic improvements from selective breeding. To balance the recovery of genetic diversity with the maintenance of an improved phenotype, a well-managed breeding plan is essential. Optimum contribution selection with a restriction on inbreeding is useful for small breeding populations to maximize genetic gain and simultaneously manage increases in inbreeding rate (Meuwissen 1997; Fernández et al. 2011). However, the mixed-use of genomic and pedigree information for breeding value prediction and relatedness estimation will increase genomic rates of inbreeding more than expected, whereas desired inbreeding control can be achieved if both estimations are made using the same information (i.e., genomic base only or pedigree base only); therefore, mixed use of this information should be avoided (Sonesson et al. 2012). To maximize the efficiency of assisted gene flow for aquaculture breeding, further research is needed into the selection of source populations, individuals, and breeding strategies.

Although such limitations needed to be addressed in future studies, our study demonstrated the feasibility of gene flow with genomic selection for genetic management of small populations. This will help the sustainable management of Japanese aquaculture populations of coho salmon. As small-scale aquaculture production is crucial for global food security and local economies (Garlock et al. 2022; Dam Lam et al. 2022), the technologies that allow improvements in genetic characteristics and management of genetic health of such populations simultaneously will significantly enhance the sustainability and resilience of aquaculture, contributing to the global sustainable developmental goals (SDGs) (Sonesson et al. 2023). Genome-assisted gene flow, as utilized in this study, represents onesuch promising technology.

## Supplementary Information

Below is the link to the electronic supplementary material.

Supplementary file1 (XLSX 171 KB)

## Data Availability

Sequence data have been deposited in DNA Data Bank of Japan (DDBJ) with the BioSample Submission ID: SSUB031092. Other data is provided within supplementary information file.
